# Immunohistochemical and qPCR Detection of SARS-CoV-2 in the Human Middle Ear Versus the Nasal Cavity: Case Series

**DOI:** 10.1007/s12105-021-01378-6

**Published:** 2021-08-28

**Authors:** Arwa Kurabi, Kwang Pak, Adam S. DeConde, Allen F. Ryan, Carol H. Yan

**Affiliations:** 1 Department of Surgery/ Division of Otolaryngology- Head and Neck Surgery, University of California, San Diego, USA; 2 Department of Neuroscience, University of California, San Diego, USA; 3 San Diego VA Healthcare System, San Diego, CA USA; 4 University of California San Diego, School of Medicine, 9500 Gilman Drive, La Jolla, CA 92093-0666 USA

**Keywords:** COVID-19, Coronavirus, SARS-CoV-2, Middle ear, Nasal cavity, qPCR, Immunohistochemistry

## Abstract

Viral infections have already been implicated with otitis media and sudden sensorineural hearing loss. However, the pathophysiology of COVID-19 as it relates to otologic disorders is not well-defined. With the spread of SARS-CoV-2, it is important to evaluate its colonization of middle ear mucosa. Middle ear and nasal tissue samples for quantitative RT-PCR and histologic evaluations were obtained from post-mortem COVID-19 patients and non-diseased control patients. Here we present evidence that SARS-CoV-2 colonizes the middle ear epithelium and co-localizes with the primary viral receptor, angiotensin-converting enzyme 2 (ACE2). Both middle ear and nasal epithelial cells show relatively high expression of ACE2, required for SARS-CoV-2 entry. The epithelial cell adhesion molecule (EpCAM) was use as a biomarker of epithelia. Furthermore, we found that the viral load in the middle ear is lower than that present in the nasal cavity.

## Introduction

COVID-19 caused by the severe acute respiratory syndrome coronavirus 2 (SARS-CoV-2) has been linked to acute otitis media (OM) [1], as well as longer term otologic sequelae including hearing loss and tinnitus [2]. In a survey of admitted COVID-19 patients, 1 in 10 reported changes in their hearing [3]. Other respiratory viruses are known to cause OM through increased susceptibility to bacterial OM or, less commonly, direct viral infection of the middle ear (ME) [4, 5]. The pathophysiology of COVID-19 associated otologic disorders has not yet been elucidated.

Although the nasal cavity is a known viral entry site, there is little data as to whether the mucosal epithelium lining the ME is infected and if so, the resulting clinical implications. One prior qPCR study demonstrated the presence of SARS-CoV-2 RNA in the mastoid and ME in autopsy specimens [6], but specific infected tissue tropism was unknown. More information is needed regarding ME colonization, viral distribution, the potential ME effects of COVID-19 and its relationship to viral infection in the nasal cavity.

In this case series, we examined the ME and nasal septal tissue from post-mortem COVID-19 patients for evidence of SARS-CoV-2 colonization. We also evaluated these tissues for expression of the primary viral receptor, angiotensin-converting enzyme 2 (ACE2).

## Materials and Methods

### Subjects

ME and nasal cavity mucosal samples were obtained post-mortem from six COVID-19 patients (P1-6) with PCR-confirmed SARS-CoV-2. The median age at death was 65 years (range, 44 to 91 years), and the time from COVID-19 symptom onset to autopsy ranged from 6 to 37 days (see Table 1 for further clinical details). All subjects had acute respiratory distress syndrome (ARDS) and hearing loss was present in two of the six subjects (P1 and P5). PCR-confirmed, COVID-19 negative patients (P7-10) served as controls with tissue obtained during routine otolaryngologic surgeries unrelated to middle ear or nasal mucosa abnormalities. The use of human tissues was reviewed and approved by the Institutional Review Board of the University of California San Diego School of Medicine. (IRB#191,951 & 151,473).

**Table 1:** Clinical and demographic information from patients utilized in this study

Patient ID	Gender	Age (years)	Days between diagnosis and death	Days between symptoms and death	ARDS	Acute renal failure	HTN	Diabetes	Hearing loss
					Hospitalization		PMH		
COVID-19									
1	F	88	17	18	Y	N	Y	N	Y
2	M	65	35	37	Y	Y	N	N	N
3	M	57	28	28	Y	Y	N	Y	N
4	F	44	6	8	Y	Y	N	N	N
5	F	91	3	6	Y	N	N	N	Y
6	M	65	28	28	Y	Y	N	Y	N
Control									
7	M	35	–	–	–	–	N	N	N
8	F	44	–	–	–	–	Y	Y	N
9	F	39	–	–	–	–	N	N	N
10	M	63	–	–	–	–	Y	N	Y

*N* No, *Y* yes

### Tissue Procurement and Processing

ME and nasal septal mucosa were harvested endoscopically by attending otolaryngologists (CHY, ASD) within 3 h post-mortem from COVID-19 patients or at time of otolaryngologic surgery for non-COVID-19 control cases (P7, P8, P9, P10). Given the limited tissue amount, samples were utilized for either RT-qPCR analysis (P1, P2, P3, P7, P8, P9, P10) or histological evaluation (P4, P5, P6, P7, P8). Samples were placed in trizol for RNA analysis or fixed in 4% paraformaldehyde for 24 h followed by paraffin embedding. Blocks were cut into 8 µm sections for hematoxylin and eosin (H&E) and all immunofluorescence staining.

### COVID-19 qPCR

Quantitative RT-PCR for SARS-CoV-2 detection was used per the US Centers for Disease Control panel assay [7]. COVID-19 primers (N1, N2, and N3 -GENEWIZ) plus human GAPDH gene (Qiagen) were used to assess all clinical samples using the StepOnePlus PCR cycler system (Applied Biosystems). A quantitative synthetic SARS-CoV-2 RNA: Spike 3’ template (ATCC-VR3276SD) was used as a positive control template. Total RNA was extracted by Trizol/chlorophorm protocol (Invitrogen) and reverse transcribed using SuperScript-III (Invitrogen). The relative expression was normalized to that of human GAPDH gene present using the ΔΔC_t_ method.

Efficiency of primers was determined to be 95–100% by standard curve, and melt curves were used to assure the correct amplicon size. To calculate viral load, the quantitative synthetic COVID-19 template was used to generate a standard curve for quantification [8]. All experiments were performed in triplicates per biological sample with error bars depicting standard error. Statistical significance was determined by Wilcoxon non-parametric test with p < 0.05. Samples with Ct values > 38 or undetectable were considered negative.

### Immunofluorescence

Deparaffinized and hydrated sections were heated in citrate solution (pH 6.0) for antigen retrieval (DAKO). After blocking for 1 h. using 2% BSA, sections were incubated with primary antibodies that were either from rabbit or mouse to: hEPCAM (Invitrogen 1:200), hACE-2 (Abcam 1:200), Muc5AC (Abcam, 1:200), SARS-CoV-2 Spike (Abcam, 1:100) [9] and SARS-CoV-2 nucleocapsid (Sinobiogical, 1:100) [10], overnight. Following further PBS washs, the appropriate Alexa-Fluor-conjugated secondary antibodies (Molecular Probes, Eugene, OR, 1:700) were incubated for 1 h. at room temperature, washed in PBS, mounted in Slow fade for imaging. Immunostained slides were imaged with an FSX100 microscope (Olympus) and exposure-matched pictures from negative controls compared.

## Results

**Fig. 1:**
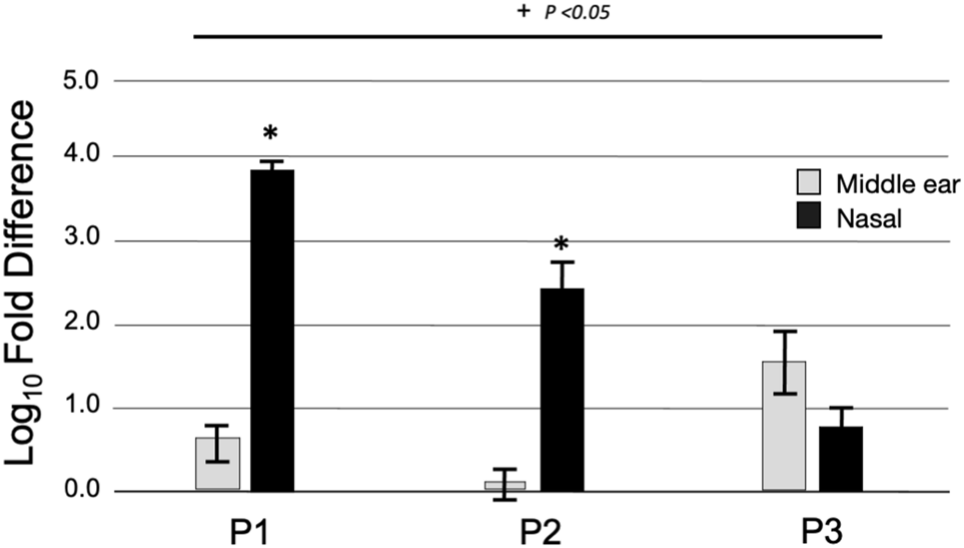
SARS-CoV-2 RNA expression in the middle ear and nasal cavity. Expression depicted as log_10_ fold difference, normalized to control COVID-19 negative tissue (P7 and P8, septal mucosa). All COVID-19 ME and nasal cavity samples demonstrated statistically higher levels of SARS-CoV-2 RNA expression compared to control except for the ME sample for P2 as indicated by ^+^(p < 0.05). A noninfectious positive control template yielded strong positive results in each assay (data not shown). For subjects P1 and P2, the viral loads in the nasal cavity were higher than those in the ME, as indicated by *(p < 0.05)

SARS-CoV-2 RNA expression in the ME and the nasal cavity are overall increased in COVID-19 subjects compared to non-infected patients control tissue, with higher expression in the nasal cavity compared to the ME (Fig. 1). ME mucosa from individuals P1 and P3 was positive for SARS-CoV-2 RNA by RT-qPCR with cycle thresholds ranging from 29–33. Meanwhile, in the nasal tissue, all three samples were positive for SARS-CoV-2 with cycle thresholds ranging in 23–32. The viral loads (Table 2) in the nasal cavity were significantly higher than in the ME for subjects P1 (2 × 10^5^ vs. 2 × 10^2^ copies /µl, p < 0.05) and P2 (1 × 10^3^ copies /µl vs. ME COVID-19 negative). There was no statistical difference in viral load for subject P3 (3.8 × 10^2^ vs. 1 × 10^2^ copies /µl, ME vs. nasal cavity).

**Table 2:** Quantitative RT-PCR data for SARS-CoV-2 detection in the nasal cavity and middle ear samples

Patient ID	Nasal		Middle ear	
	Ct value	Viral load copies /µl	Ct value	Viral load (copies /µl
1	23.2	2.4 × 10^5^	31.2	2.2 × 10^2^
2	27.3	1.0 × 10^3^	38.0	–
3	32.5	9.3 × 10^1^	29.6	3.8 × 10^2^
4	ND		ND	
5	ND		ND	
6	ND		ND	
7	> 38	–	ND	–
8	> 38	–	ND	–
9	> 38	–	> 38	–
10	> 38	–	> 38	–

ND stands for not determined. Samples with Ct values > 38 were considered negative

**Fig. 2:**
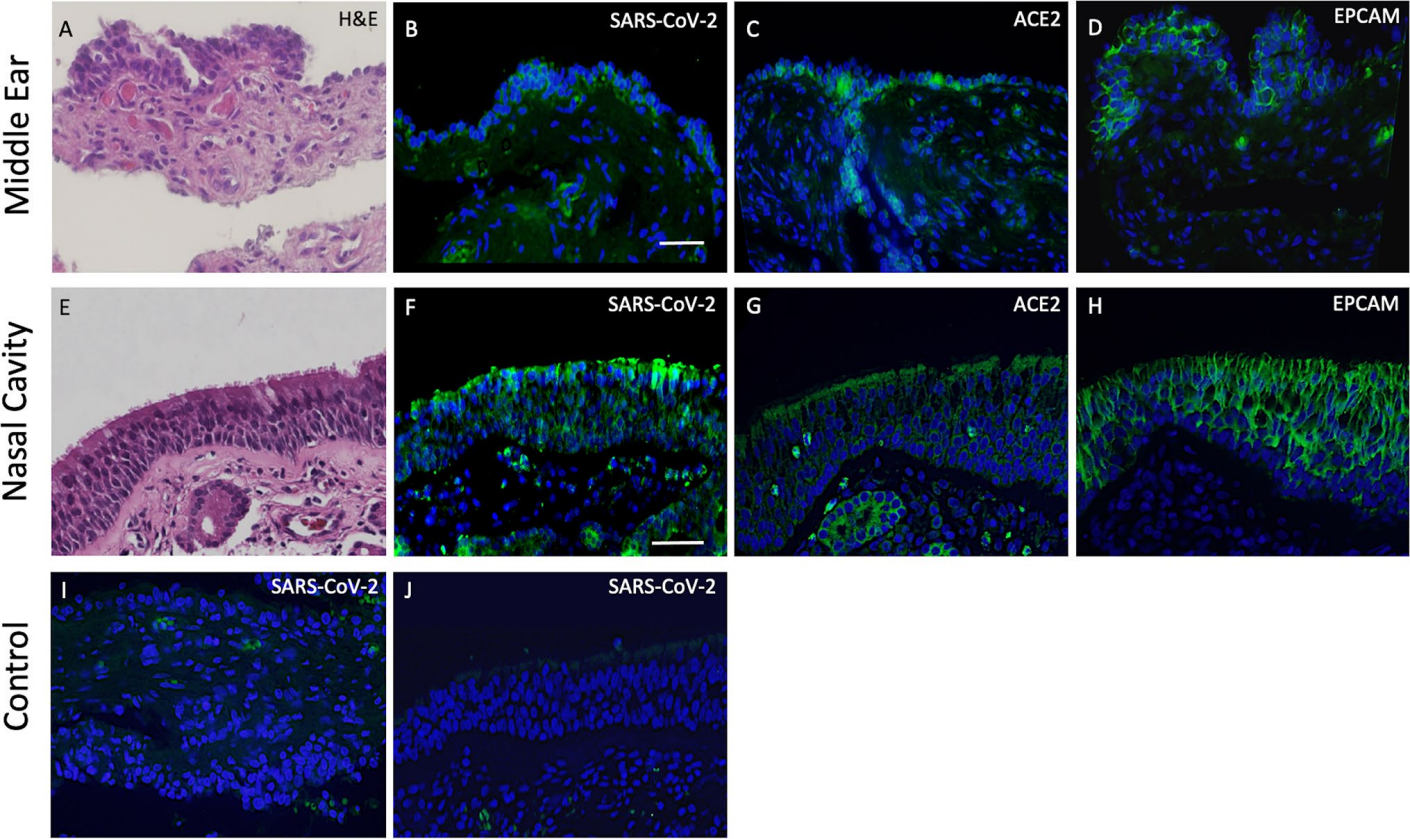
H&E and immunofluorescence staining of middle ear and nasal cavity tissues from COVID-19 and uninfected individuals. Histology **A**, **E** plus immunohistochemical localization of SARS-CoV-2 in epithelia of COVID-19 patients (**B**, **F**). Expression of ACE2 (**C**, **G**) was detected on surface epithelium identified by EPCAM (**D**, **H**). No SARS-CoV-2 staining was seen in COVID-19 negative tissues (**I**, **J**), although RBCs showed autofluorescence

Histological evaluations of tissue from patients P4, P5 and P6 all showed that expression of SARS-CoV-2 was found in both the ME and nasal septum which co-localized with ACE2 expression (Fig. 2). Expression of ACE2 was detected on surface epithelium identified by EPCAM. Both ME and nasal epithelial cells showed relatively high expression of ACE2 required for SARS-CoV-2 entry. Viral staining was not detected in COVID-19 negative and control ME and nasal samples. Paraffin sections were labeled with DAPI nuclear staining (blue) and the indicated Alexa 488-conjugated secondary antibodies (green) in representative sections in Fig. 2.

## Discussion

We demonstrate concomitant viral presence in the ME and nasal cavity, suggesting systemic infection of SARS-CoV-2 in the head and neck region. In the ME viral load due to other upper respiratory tract (URT) viral infections often varies with a range between (10^1^–10^4^ copies/µl) [11, 12] with higher viral loads typically associated with increasing URT infection severity [12, 13]. Looking at COVID-19 studies in other organ systems, viral loads have been reported in the range of 10^1^–10^5^ copies/µL in gut and 10^1^–10^3^ copies/µL in blood [14]. Hence SARS-CoV-2 colonizes the ME at comparable viral loads in our preliminary findings although additional samples are required to strengthen these comparisons. Moreover, given the lower viral loads observed in the ME tissue compared to the nasal cavity, we hypothesize that ME viral entry occurs via the eustachian tube, as previously demonstrated by other viral URT infections that lead to otitis media [5, 15]. However, circulation-mediated viral entry is also possible. Both the nasal cavity and ME epithelium express ACE2, the receptor known to interact with the SARS-CoV-2 spike protein for cell entry.

ME viral infection has been previously shown to contribute to otologic pathology and symptom manifestations such as OM and conductive hearing loss [5]. It is unclear if SARS-CoV-2 may further damage the auditory sensorineural system or if the COVID-19 symptoms of hearing loss and tinnitus may reflect the ototoxic effects of antiviral medications or immune mediators such as cytokines. However, the presence of viral loads in the ME could lead to direct inner ear SARS-CoV-2 infections. It is also possible that the virus triggers inner ear autoimmunity, known to produce hearing loss [16]. In our sample size of six COVID-19 patients, two had baseline hearing loss and none had reported COVID-19 related hearing loss. However, all suffered from abrupt clinical deterioration with ARDS and passed from COVID-19 complications, and thus milder otologic symptoms may have been overlooked. Further case studies on long-haul sequelae of COVID-19 survivors would shed more light on long-term effects. Our study is limited by its small sample size and future research regarding potential effects of COVID-19 on the ME and otologic symptomatology are warranted.

## Data Availability

Any data is available upon request.
